# Measurement of hydroxychloroquine in blood from SLE patients using LC-HRMS—evaluation of whole blood, plasma, and serum as sample matrices

**DOI:** 10.1186/s13075-020-02211-1

**Published:** 2020-06-01

**Authors:** Henrik Carlsson, Karin Hjorton, Sandy Abujrais, Lars Rönnblom, Torbjörn Åkerfeldt, Kim Kultima

**Affiliations:** 1 Department of Medical Sciences, Clinical Chemistry, Uppsala University, Uppsala University Hospital, Entrance 61, 3rd floor, Dag Hammarskjölds Väg 18, SE-751 85 Uppsala, Sweden; 2 Department of Medical Sciences, Rheumatology, Uppsala University, Uppsala, Sweden

## Abstract

**Background:**

Hydroxychloroquine (HCQ) is the standard of care in the treatment of systemic lupus erythematosus (SLE), rheumatoid arthritis (RA), and other inflammatory rheumatic diseases and potentially for the treatment in COVID-19 patients. Determination of HCQ for therapeutic drug monitoring (TDM) can be performed in whole blood (WB), serum, and plasma. Direct comparisons of WB, serum, and plasma levels of HCQ in patients with SLE have not previously been reported. We describe a method for the determination of HCQ in human blood using liquid chromatography-high-resolution mass spectrometry (LC-HRMS) and compare the suitability of the three sample matrices.

**Methods:**

A method for the determination of HCQ in human blood using LC-HRMS was developed, validated, and applied for the determination of HCQ levels in WB, serum, and plasma from 26 SLE patients. The reproducibility of the method, in the three matrices, was evaluated using quality control samples and repeated preparations and measurements of patient samples. The performance of the developed method for HCQ measurement in serum was further evaluated by comparison with two previously reported extraction methods.

**Results:**

The performance of the presented method demonstrated high accuracy and precision. A large range of HCQ concentrations was observed for the SLE patients in all three matrices (WB, serum, and plasma). The mean levels in WB were approximately two-fold the levels in serum and plasma (813 ng/mL compared to 436 ng/mL and 362 ng/mL, respectively). Spiked quality controls showed high reproducibility for all matrices (coefficient of variation, CV, approx. 5%), whereas in patient samples, equally high-precision was only found using WB as the matrix (CV 3%). The CV for serum and plasma was 14% and 39%, respectively. Two alternative methods applied to serum samples did not demonstrate improved precision.

**Conclusions:**

A LC-HRMS method for the measurement of HCQ in human blood was developed and validated. Whole blood was found to be the superior sample matrix in terms of sample reproducibility. Thus, whole blood samples should be used for HCQ analysis when patients are monitored for HCQ treatment effects. The assay is in clinical use to monitor levels of HCQ in patients.

## Introduction

Systemic lupus erythematosus (SLE) is a chronic autoimmune disease affecting predominantly women, with an up to 9:1 ratio in the young and middle-aged [1]. The clinical phenotype varies from milder forms, typically with skin rash and arthritis, to severe, such as lupus nephritis or neuropsychiatric lupus, with a high risk of persistent organ damage, caused by either the inflammation or its treatment. Despite the variable clinical phenotype, antimalarials such as hydroxychloroquine (HCQ) and chloroquine phosphate are standard of care in SLE [2, 3], rheumatoid arthritis (RA) [4], or other inflammatory rheumatic diseases [5]. Recently, they have been suggested for treatment in COVID-19 patients [6]. Their benefits include improved survival, significant reduction of SLE flare risk as well as organ damage, delayed onset of disease, and less thrombotic events [7]. Hydroxychloroquine is more commonly used due to a more favorable side effect profile than chloroquine phosphate [8]. Studies have shown a correlation between low blood concentrations of HCQ and increased disease activity in SLE, as well as risk of flare [9, 10] and premature birth [11]. Drug concentrations vary considerably between individuals despite same dosing regimen and are influenced by factors like weight/BMI, glomerular filtration rate [12], non-adherence to therapy [13], and possibly genetic variations [14]. Furthermore, flares occur in some patients despite seemingly adequate treatment, the reasons for which, in many cases, are unclear. Since quinolones accumulate in pigmented tissue, a risk of permanent visual impairment due to eye deposits exists. As the risk increases with cumulative dose, approaching 10–20% after 16–20 years [15, 16], the recommendations from the American Academy of Ophthalmology are a maximum dosage of 5 mg/kg real body weight and day [17, 18].

Determination of HCQ and its metabolites can be performed in whole blood (WB), serum, and plasma. Traditionally, HCQ levels have been determined in WB [19], whereas some recent studies have reported measuring HCQ in serum [20, 21]. One single study reported the levels in WB to be five times higher than those in plasma [22]. Direct comparisons of WB, serum, and plasma levels of HCQ in patients with SLE have not been reported.

The majority of HCQ measurements in human biofluids use liquid chromatography (LC) coupled to triple quadrupole (QqQ) mass spectrometry (MS), where the analysis is performed in the multiple reaction monitoring mode [20, 23–26]. There are no methods presented using LC-high-resolution mass spectrometry (LC-HRMS). LC-HRMS is advantageous since it allows the measurement of ions with high resolution and accuracy, enabling highly efficient filtering of ions for removal of interferences and background noise which may improve on detection and quantification limits.

The specific aims of the current study were to (i) establish and validate a method using LC-HRMS for the determination of blood HCQ concentration in HCQ/Plaquenil-treated SLE patients; (ii) determine the concentration of HCQ in WB, serum, and plasma and evaluate the reproducibility for the different matrices in matched samples; and (iii) perform a full validation of the LC-HRMS method for the determination of HCQ concentrations, to be used in a clinical setting for TDM.

## Methods

### Chemicals

Acetonitrile (ACN, ≥ 99.9%, MS grade, VWR Chemicals, Leuven, Belgium), formic acid (FA, ACS reagent grade), and ammonium formate (MS grade) from Merck (Darmstadt, Germany) and dimethyl sulfoxide (DMSO, HPLC grade) from Sigma-Aldrich (St. Louis, MO, USA) and water using Advantage A10 Milli-Q system (Merck Millipore, Burlington, MA, USA). Hydroxychloroquine sulfate (1.0 mg/ml in methanol as free base) was obtained from LGC standards (Teddington, England) and hydroxychloroquine-d4 sulfate from Toronto Research Chemicals (North York, ON, Canada).

### Ethical approval

All participants provided written informed consent, and the study was approved by the Regional Ethical Board of Uppsala (Dnr: 2019-02031).

### Samples

Whole blood, serum, and plasma were jointly collected from 26 SLE patients at the Uppsala University Hospital. Pooled WB, serum, and plasma with no detectable HCQ were obtained from Uppsala University Hospital and used for blanks, calibrators, and quality controls. Whole blood was collected freshly from three additional SLE patients at the Uppsala University Hospital to evaluate the long-term stability of HCQ in WB. For the collection of WB and plasma, vacutainer tubes with EDTA as the anticoagulant were used, and for the collection of serum, tubes with no gel were used. The samples were stored at -80 °C until analysis.

### Patients

All patients fulfilled ≥ 4 of the classification criteria for SLE according to the 1982 American College of Rheumatology (ACR) or the 2012 Systemic Lupus International Collaborating Clinics (SLICC) [27, 28]. Patients were 49.5 years old (median) with a median disease duration of 10.5 years, and 81% were women (Table 1). All patients were administered HCQ in the form of 200 mg Plaquenil® tablets. Out of the 26 patients, 14 were prescribed 1400 mg (1 tablet daily), nine patients 2000 mg (200 mg/day, 4 days; 400 mg/day, 3 days per week), and two patients 2800 mg Plaquenil weekly (400 mg/day). Furthermore, one patient had stopped taking Plaquenil but was included in the study as a control subject. A majority of patients (80%) had low Systemic Lupus Erythematosus Disease Activity Index (SLEDAI 0–6) [29] at the time of blood sampling, 58% were on at least one additional disease-modifying antirheumatic drug (DMARD), and the median prednisone dose was 1.25 mg daily.

**Table 1:** Characteristics of HCQ-treated SLE patients

Patient characteristics (***n*** = 26)	
Females, *n* (%)	21 (81)
Age years, median (range)	49.5 (20–79)
Disease duration years, median (range)	10.5 (1–30)
Number of ACR criteria, median (range)	5 (4–9)
Number of SLICC criteria, median (range)	7 (4–12)
SLICC/ACR damage index, median (range)	0 (0–4)
SLEDAI at blood sampling, median (range)	2 (0–18)
Creatinine (μmol/L), median (range)	70 (41–178)
Alanine aminotransferase (μkat/L), median (range)	0.43 (0.16–0.87)
BMI^a^, median (range)	25 (18.4–35.3)
HCQ dose 2800 mg/week, *n* (%)	2 (8)
HCQ dose 1800–2000 mg/week, *n* (%)	9 (35)
HCQ dose 1400 mg/week, *n* (%)	14 (54)
HCQ dose 0 mg/week, *n* (%)	1 (4)
Prednisone dose, mg/day median (range)	1.25 (0–25)
Azathioprine, *n* (%)	7 (29)
Mycophenolate mofetil, *n* (%)	2 (8)
Methotrexate, *n* (%)	2 (8)
Intravenous immunoglobulins, *n* (%)	2 (8)
Rituximab, *n* (%)	2 (8)
Belimumab, *n* (%)	1 (4)
No other DMARDs, *n* (%)	11 (42)

^a^BMI available for 13 out of 26 patients

*ACR* American College of Rheumatology, *SLICC* Systemic Lupus International Collaborating Clinics [27, 28], *SLEDAI* Systemic Lupus Erythematosus Disease Activity Index [29], *DMARD* disease-modifying antirheumatic drug

### Calibration curve and quality controls

Calibration standards at seven levels ranging from 8.3–6075 ng/mL (8.3, 25, 75, 225, 675, 2025, 6075 ng/mL) and quality control (QC) standards at three levels (lower limit of quantification (LLOQ) 8.3 ng/mL; low (QCL) 25 ng/mL; high (QCH) 4556.3 ng/mL) were prepared in 95:5 ACN to DMSO (v/v). An internal standard (IS) solution of hydroxychloroquine-d4 (HCQ-d4) was prepared at a concentration of 80 ng/mL in 95:5 ACN to DMSO (v/v). These solutions were stored at -20 °C. The LLOQ was defined as the lowest calibrator (8.33 ng/mL) following evaluation regarding accuracy and precision at this concentration level. Calibration standards and QC samples were prepared freshly for each analytical run. Weighted regression (1/*x*^2^) was used for the calibration curves using TraceFinder 4.1 from Thermo Scientific (Waltham, MA, USA).

### Sample preparation

All samples were frozen at -80° for at least 24 h followed by thawing to ensure hemolysis of red blood cells. For each sample, 50 μL (WB, serum, or plasma) was mixed with 20 μL of IS. Proteins were precipitated by the addition of 130 μL 95:5 ACN to DMSO, followed by vortexing and mixing for 5 min and centrifugation at 10000×*g* for 10 min. One hundred microliters of the supernatant was diluted with 900 μL H_2_O. For calibrators and QC samples, 50 μL of the blank sample was used and 50 μL of either calibrator or QC working solution was added, followed by 20 μL of IS and 80 μL 95:5 ACN to DMSO prior to mixing and centrifugation as for the samples described above.

### Liquid chromatography-mass spectrometry analysis

Fifty-microliter sample was injected on a reversed phase UPLC column (Accucore C18 50 × 2.1 mm, 2.6 μm, Thermo Scientific) using an Ultimate 3000 HPLC system (Thermo Scientific) interfaced to a high-resolution hybrid quadrupole Q Exactive Orbitrap MS (Thermo Scientific). A gradient was applied using the mobile phases 10 mM ammonium formate and 0.4% FA in H_2_O (mobile phase A) and 0.4% FA in ACN (mobile phase B): 5–25% B for 2 min followed by washing at 95% B for 1 min and re-equilibration at 5% B for 1 min, with a flow rate of 0.5 mL/min (1.0 mL/min during the washing step). The HRMS was operated in positive ionization mode with the following parameters: sheath gas flow rate 50, auxiliary gas flow rate 8, sweep gas flow rate 3, spray voltage 4.5 kV, capillary temperature 320 °C, S-lens RF level 100, and auxiliary gas heater 500 °C. The HRMS analysis was performed using targeted single ion monitoring, with the target *m*/*z* 338 and an isolation window of 7 *m*/*z*, collecting data in the profile mode with the resolution 70,000.

### Method validation

The developed method was validated according to the guidelines for bioanalytical method validation defined by the European Medicines Agency (EMA;EMEA/CHMP/EWP/192217/2009 Rev. 1.). The validation was performed using WB as the sample matrix.

The extent of carry-over was evaluated by injecting a series of blanks following the highest calibrator (6075 ng/mL) in each analytical run during the validation, comparing the HCQ peak area in these blanks with the peak area of the calibrator at the LLOQ level. Carry-over in the blank after a high concentration injection should not exceed 20% of the LLOQ.

Accuracy and precision were evaluated within-run and between-runs by analyzing five replicates of QC samples at the LLOQ, QCL, and QCH levels on four different days. The stability of prepared samples stored in the autosampler (10 °C) was evaluated by reinjection of five replicates of QCL and QCH samples after 48 and 72 h. The stability of unprepared samples (4 h at RT) and for freezing (four cycles at -80 °C) was evaluated using seven SLE patient samples.

The long-term stability of HCQ in WB (unprepared samples) in RT and refrigerator (4 °C) was evaluated over 168 h for freshly collected samples from three SLE patients. After aliquotation (400 μL), samples were stored in RT or refrigerator and then frozen (-80 °C) at six different time points: 0 h, 8 h, 24 h, 48 h, 72 h, and 168 h (1 week). The samples were then thawed and prepared for analysis at one single occasion.

### Clinical application

Whole blood, serum, and plasma from 26 SLE patients were analyzed using the described method. Four patients were sampled twice (two at an interval of 10 days and two at an interval of 60 days), thus amounting to a total of 30 samples analyzed for the three matrices. To evaluate the reproducibility in the three matrices, WB, serum, and plasma from eight patients were prepared and analyzed on a second occasion. Five replicates of QCL and QCH were prepared in each matrix both times.

### Statistical analysis

The association between the measured HCQ levels in WB and weekly dose was modeled using linear mixed effect models [30], adjusting for age and gender. Hydroxychloroquine level was treated as dependent variable; patient, gender, and age as random effect variable; and weekly dose, as independent fixed effect variables. BMI was recorded for only 13 patients and therefore not included in the linear mixed effect model. The associations were evaluated using the likelihood ratio test. A *p* value of < 0.05 was considered statistically significant.

### Comparison with previously used protocols for extraction of HCQ from serum

Two alternative methods for HCQ extraction from serum were selected to compare their reproducibility performance with the present method: an ACN-based method developed for serum [20] and a methanol-based method originally developed for WB [24]. In brief, the volume of solvent (ACN or methanol) was increased from 150 to 200 μL (sample-to-solvent ratio from 1:3 to 1:4) and following centrifugation mobile phase A was used to acidify and dilute the supernatants. Seven serum samples, as well as five QC samples at the QCL and QCH levels, were prepared with the three methods at two occasions. The reproducibility of the protocols was compared in terms of within- and between-run precision.

## Results

### Method validation

The developed method for LC-HRMS analysis of HCQ in WB displayed desirable robustness and selectivity. The assay exhibited linearity in the range 8.3–6075 ng/mL with no interferences observed and with coefficients of determination (*R*^2^) > 0.997 for the calibration curves.

Following an injection of 6075 ng/mL HCQ (the highest calibrator), carry-over was observed at approximately 25% of the LLOQ (8.3 ng/mL). This was reduced to 5% by injecting two additional blanks (three blanks in total).

The within- and between-run accuracy and precision were within the recommended EMA guidelines, with bias < 15% in comparison with the nominal concentrations and CV < 5% at each of the three evaluated levels (LLOQ 8.3 ng/mL, QCL 25 ng/mL, and QCH 4556.3 ng/mL) (Table 2) in order to validate the accuracy and reproducibility of the measurements within the methodological range. The stability of the 48 and 72 h autosampler-stored QC samples (10 °C) agreed with the EMA guidelines (Table 2). The stability of unprepared samples at room temperature and after four repeated freeze-thaw cycles was satisfactory with an average CV < 5% when comparing measured concentrations before and after storing treatment (Table 3).

**Table 2:** Intra- and inter-day precision (CV%) and accuracy (bias%) at three QC levels

		Intra-day (*n* = 5)			Inter-day (*n* = 20)		
QC level	Nominal concentration (ng/mL)	Mean concentration ± SD (ng/mL)	CV%	Bias%	Mean concentration ± SD (ng/mL)	CV%	Bias%
LLOQ	8.3	8.1 ± 0.3	3.2	− 3.3	7.8 ± 0.4	4.9	− 6.0
QCL	25.0	23.3 ± 0.3	1.1	− 6.9	22.6 ± 0.8	3.5	− 9.5
QCH	4556.3	4563.8 ± 205.0	4.5	0.2	4432.4 ± 157.5	3.6	− 2.7

**Table 3:** Short-term stability of samples. Stability of prepared samples stored in autosampler (10 °C), and stability of patient samples at RT and following repeated freeze-thawing

	QCL (25 ng/mL) (*n* = 5)		QCH (4556.3 ng/mL) (*n* = 5)		Patient samples (*n* = 7)	
	CV%	Bias%	CV%	Bias%	CV%	Diff%
Autosampler, 48 h	5.2	3.8	4.0	− 2.5	–	–
Autosampler, 72 h	7.8	13.7	4.3	− 2.5	–	–
Room temp., 4 h	–	–	–	–	4.1	5.0
Freeze-thaw, 4 cycles	–	–	–	–	2.5	− 0.2

*Bias%* percent difference from nominal conc., *Diff%* average percent difference, measured conc. after storage subtracted from conc. prior

The stability of HCQ in WB stored at RT or refrigerator (4 °C) was high with no significant difference in the measured HCQ concentration after up to 168 h (1 week) of storage at either temperature (Table 4). The average CV of the HCQ concentrations for the three patient samples measured at the six time points were 3.5% in RT and 3.2% in the refrigerator.

**Table 4:** Long-term stability of HCQ in whole blood. HCQ concentrations in three WB samples stored up to 168 h (1 week) at either in RT or in the refrigerator (4 °C)

	Patient 1		Patient 2		Patient 3	
Hours	RT, ng/mL	Refrigerator, ng/mL	RT, ng/mL	Refrigerator, ng/mL	RT, ng/mL	Refrigerator, ng/mL
0	770		432		307	
8	778	776	403	416	304	295
24	777	755	421	411	293	307
48	754	848	410	397	284	303
72	784	760	429	391	325	295
168 (1 week)	756	780	449	410	291	305
Mean ± SD (ng/mL)	770 ± 13	781 ± 34	424 ± 17	409 ± 14	301 ± 15	302 ± 6
CV%	1.6	4.3	3.9	3.5	4.9	1.9

### Clinical applications

**Fig. 1:**
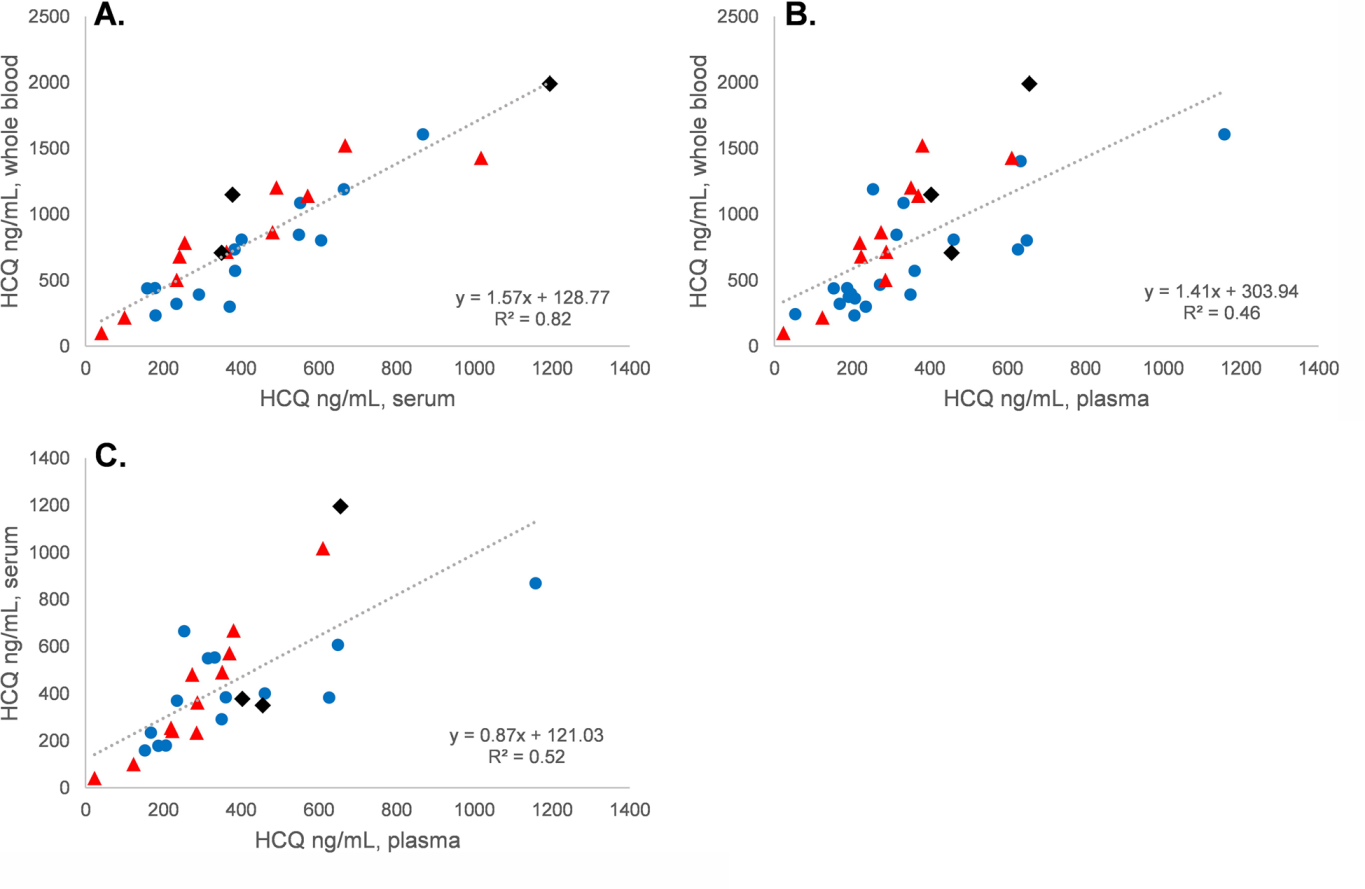
Comparison of HCQ measurements in three matrices for the same samples. **a** Whole blood versus serum. **b** Whole blood versus plasma. **c** Serum versus plasma. The markers indicate prescribed dosage of HCQ: circle = 1400 mg/week, triangle = 2000 mg/week, and diamond = 2800 mg/week

The highest levels in patients were observed in WB, followed by serum and plasma (Table 2 and Fig. 1). In three patients, no HCQ was detected (one who had terminated HCQ (0 mg/week), and two prescribed 1400 mg/week). A large range in HCQ concentrations was observed in all matrices. In patients with the 1400-mg weekly dosage and detectable HCQ (*n* = 12), the concentration range differed 7-fold (233–1606 ng/mL), 5-fold (159–868 ng/mL), and 8-fold (153–1158 ng/mL) for WB, serum, and plasma, respectively. Patients with the 2000-mg HCQ/week dosage (*n* = 9) displayed a wider concentration range, differing 16-fold (98–1522 ng/mL), 25-fold (41–1018 ng/mL), and up to 26-fold (23–611 ng/mL) for WB, serum, and plasma, respectively. The highest WB (1989 ng/mL) and serum (1194 ng/mL) levels were found in a patient prescribed 2800 mg/week (Table 5 and Fig. 1). In the four patients sampled twice, the concentration range differed 1.1- to 2.3-fold between the two occasions (Table 5).

**Table 5:** Mean, standard deviation (SD), and range of HCQ (ng/mL) measured in the three matrices. Three dose levels were prescribed (1400, 2000, and 2800 mg/week) and four patients (A–D) were sampled twice

	HCQ Conc ng/mL		
	Whole blood	Serum	Plasma
Mean and range. All doses (*n* = 25^a^)	813 ± 466 (n.d.^b^–1989)	436 ± 270 (n.d.^b^–1194)	362 ± 224 (n.d.^b^ − 1158)
Mean and range. Dose 1400 mg/week (*n* = 14)	697 ± 395 (n.d.–1606)	416 ± 209 (n.d.–868)	390 ± 271 (n.d.–1158)
Mean and range. Dose 2000 mg/week (*n* = 9)	832 ± 461 (98–1522)	406 ± 281 (41–1018)	287 ± 152 (23–611)
Mean and range. Dose 2800 mg/week (*n* = 2)	1282 ± 651 (709–1989)	641 ± 480 (350–1194)	505 ± 133 (404–656)
Patient A, 1st and 2nd sample, 10 days between samplings (2000 mg/week)	503, 216	235, 100	286, 124
Patient B, 1st and 2nd sample, 10 days between samplings (2000 mg/week)	680, 717	242, 363	223, 288
Patient C, 1st and 2nd sample, 60 days between samplings (1400 mg/week)	390, 298	292, 371	350, 235
Patient D, 1st and 2nd sample, 60 days between samplings (2800 mg/week)	709, 1147	350, 378	456, 404

^a^The patient with no HCQ administered (0 mg/week) is excluded from this table

^b^*n.d*. not detected; excluded from the calculation of mean and SD

The correlation between WB and serum was acceptable (*R*^2^ = 0.82), but comparisons involving plasma measurements showed unacceptably high variability (*R*^2^ = 0.46 for WB vs plasma and *R*^2^ = 0.52 for serum vs plasma). The average relation between HCQ levels in the different matrices were for WB:serum:plasma 1:0.54:0.44. The accuracy and precision of QC samples at the QCL and QCH levels were high in all three matrices (CV and bias < 5%).

**Fig. 2:**
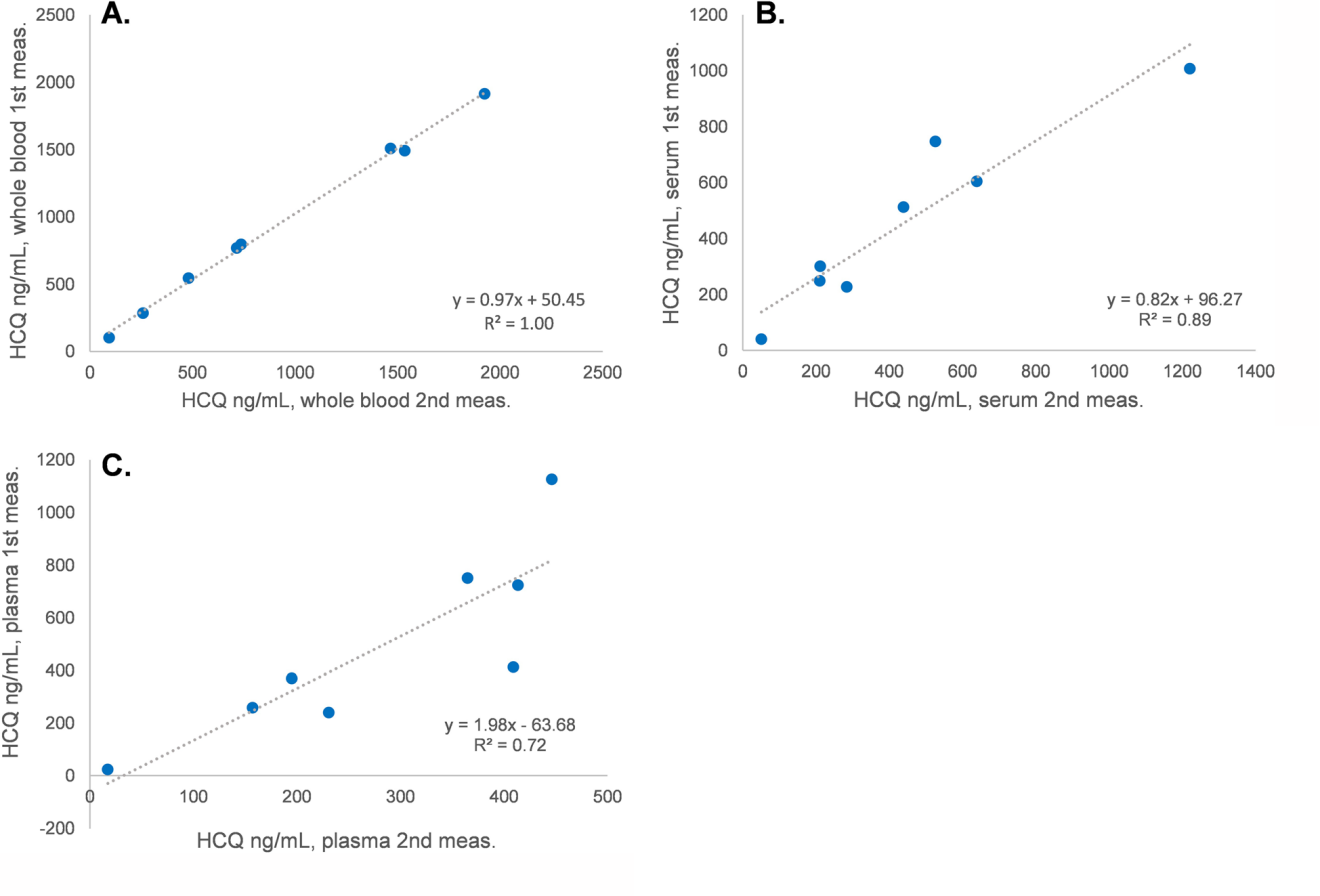
Reproducibility testing. In order to evaluate the reproducibility of the method in the three matrices, eight patient samples were processed and analyzed on two occasions. **a**, **b**, and **c** shows the results for whole blood, serum, and plasma, respectively. Reproducibility was high in WB, intermediate in serum, and low in plasma

The variation between the three matrices was unexpectedly high. To evaluate the reproducibility of the sample protocol, eight patient samples were extracted and analyzed at two separate occasions. A high reproducibility was observed for WB (*R*^2^ = 1.0, CV 3%), whereas the reproducibility for serum was intermediate (*R*^2^ = 0.89, CV 14%) and low for plasma (*R*^2^ = 0.72, CV 39%). In contrast, the reproducibility of the QC sample was high (CV 3–5%) in all matrices (Fig. 2 and Table 6).

**Table 6:** Reproducibility testing. To evaluate the reproducibility of the protocol used, eight patient samples were extracted and analyzed at two separate occasions for all three matrices. Five QC samples (QCL and QCH) were prepared on both occasions. The reproducibility of the QC samples was high in all matrices and in patients’ samples measuring WB

	Whole blood	Serum	Plasma
Mean (ng/mL)^a^	915	455	384
SD (ng/mL)^b^	27	65	148
CV (%)^c^	3	14	39
QCL (25 ng/mL), CV%	4	4	5
QCH (4556.3 ng/mL), CV%	3	4	4

^a^Mean of all measurements, eight samples measured twice

^b^Mean of the SD from comparing two measurements of the same sample, reflects reproducibility

^c^Mean of the CV from comparing two measurements of the same sample, reflects reproducibility

### Comparison with other protocols for extraction of HCQ from serum

In the repeated patient sample preparation and measurements, we found the CV to be high in serum and plasma compared to WB. In order to evaluate whether alternative extraction methods could improve the reproducibility observed for serum, the presented extraction protocol was modified to resemble that of Mok et al. [20] and Soichot et al. [24]. Seven patient serum samples were prepared using the three different protocols on two occasions. Although the QC sample reproducibility was consistently high across the three protocols (CV 2–4%), none of the alternatives improved reproducibility in serum patient samples (CV 15–17%) (Table 7).

**Table 7:** Evaluation of alternative protocols for HCQ levels in serum. Seven patient serum samples were prepared with the present method as well as the two alternative methods on two occasions. All methods showed high reproducibility for the measurement of HCQ in QC samples, but no method showed improved reproducibility for patient samples

Method	Alt. Method 1^a^ CV%	Alt. Method 2^b^ CV%	Current method CV%
Samples (*n* = 7)	16	17	15
QCL (*n* = 5)	2	3	4
QCH (*n* = 5)	3	4	4

^a^Based on the method by Mok et al. [20], using acetonitrile for precipitation

^b^Based on the method by Soichot et al. [24], using methanol for precipitation

Measurements of HCQ in WB were found to be superior to serum and plasma. The association between HCQ levels and weekly dose was modeled using the linear mixed effect model. There was a statistically significant (*p* < 0.05) association between HCQ levels in WB and weekly dose, but not in serum or plasma in this limited size study.

## Discussion

Here, we present a fast and reproducible method for the determination of HCQ concentrations in blood from SLE patients using LC-HRMS. The method which is based on a total chromatographic time of four minutes shows good linearity in the range of 8.3 to 6075 ng/mL and high reproducibility in spiked quality controls for plasma, serum, and WB matrices, respectively. In patient samples, however, high reproducibility could only be achieved measuring the HCQ concentration in WB. The reproducibility in serum was acceptable, yet in plasma unacceptably low. The superior reproducibility in WB is in line with a previous small pharmacokinetic study where plasma data were reported to be more variable than WB [22].

To further evaluate the reproducibility in patient samples, the current protocol was modified to resemble the protocols of Mok et al. [20] and Soichot et al. [24]. Reproducibility for QC samples was satisfactory for all three protocols. However, none of the methods showed any significant improvement with respect to serum patient samples (CV 15–17%), compared to WB (3%). Previous methods for HCQ analysis using LC-MS have been evaluated using QC samples, and the reproducibility of patient samples was not evaluated [20, 23–26]. Hydroxychloroquine is a drug with a plasma protein binding degree estimated to 50% [31], and it has been speculated that WB is favorable because of the technical challenges in separating blood cells and platelets from plasma [32].

These patients, who were on a stable dose of Plaquenil (> 6 months), and thus expected to have reached equilibrium concentrations [33, 34], displayed HCQ levels in WB approximately twice the levels detected in both serum and plasma. This ratio is smaller compared to previous findings, reporting a WB-plasma ratio of approximately 5:1 [22]. Hydroxychloroquine concentrations vary considerably between individuals with the same dosing regimen. For example, in WB, there was up to 16-fold difference between individuals (2000 mg HCQ/week). This high inter-individual difference has also been reported by others [9, 12, 32]. There was also an up to 2.3-fold difference (in WB) between the occasions in patients that were sampled twice (*n* = 4), with no obvious connection to the time between sampling occasions (two at a 10-day interval and two at a 60-day interval). These observations point to the fact that HCQ concentrations cannot be predicted only based on the prescribed dose [13, 35].

The high stability of HCQ in WB, with no significant decrease in concentration after prolonged storage up to 168 h, allows for collection of samples and transportation without the need of cooling or freezing. Importantly, however, samples need to be frozen at -80° for 24 h followed by thawing to ensure hemolysis of red blood cells. Shorter freezing period at -80° may be sufficient, but has not been evaluated in the current study.

In this very limited size study, intended for the evaluation of most suitable matrix for the determination of HCQ, we found a significant association between the patient’s weekly dose and HCQ levels in WB, in contrast to serum or plasma levels. Other factors including age, gender, and genetically determined drug metabolism may also influence patients’ HCQ levels in blood [12, 14].

Hydroxychloroquine measurements in routine care can help clinicians motivate patients to improve treatment adherence, and to accordingly adjust doses, avoiding unnecessary addition of potentially more toxic therapy. Monitoring HCQ concentrations could also help minimize the risk of long-term side effects such as retinopathy, as recently demonstrated that HCQ blood levels predicted later hydroxychloroquine retinopathy [16]. The optimal concentration levels to balance these aims may require further evaluation [19].

## Conclusions

The presented LC-HRMS method for the quantification of HCQ, validated using whole blood as the preferred sample matrix, was found to be superior to using serum and plasma. Hydroxychloroquine concentrations vary considerably between individuals with the same dosing regimen. The performance of the method meets the criteria defined by the EMA for method evaluation and is well suited for monitoring HCQ levels in SLE patients as well as other patient groups prescribed this drug. The assay is in use at the laboratory at the Uppsala University Hospital in Sweden to monitor levels of HCQ in patients. The laboratory is accredited by SWEDAC (SS-EN ISO 15189) to deliver results on patient samples for clinical care.

## Data Availability

The datasets used and/or analyzed during the current study are available from the corresponding author on reasonable request.

## Abbreviations

ACN: Acetonitrile
DMSO: Dimethyl sulfoxide
EMA: European Medicines Agency
FA: Formic acid
HCQ: Hydroxychloroquine
HRMS: High-resolution mass spectrometry
LC: Liquid chromatography
LLOQ: Lower limit of quantification
MeOH: Methanol
MS: Mass spectrometry
SLE: Systemic lupus erythematosus
TDM: Therapeutic drug monitoring
QC: Quality control
QCL: Quality control with low concentration
QCH: Quality control with high concentration
WB: Whole blood
